# YgiM may act as a trigger in the sepsis caused by *Klebsiella pneumoniae* through the membrane-associated ceRNA network

**DOI:** 10.3389/fgene.2022.973145

**Published:** 2022-09-23

**Authors:** Mingxiao Han, Zhihao Chen, Ping He, Ziyuan Li, Qi Chen, Zelei Tong, Min Wang, Hong Du, Haifang Zhang

**Affiliations:** ^1^ Department of Clinical Laboratory, The Second Affiliated Hospital of Soochow University, Suzhou, China; ^2^ Department of Orthopedics, The Second Affiliated Hospital of Soochow University, Suzhou, China; ^3^ Department of Musculoskeletal Oncology, Sun Yat-sen University Cancer Center, Guangzhou, China; ^4^ State Key Laboratory of Oncology in Southern China, Collaborative Innovation Center of Cancer Medicine, Guangzhou, China; ^5^ Department of Clinical Laboratory, Sichuan Province Science City Hospital, Chengdu, China

**Keywords:** sepsis, *ygiM*, *Klebsiella pneumoniae*, peroxisome, miRNA, mRNA, ceRNAs network

## Abstract

Sepsis is one of the diseases that can cause serious mortality. In *E. coli*, an inner membrane protein YgiM encoded by gene *ygiM* can target the eukaryotic peroxisome. Peroxisome is a membrane-enclosed organelle associated with the ROS metabolism and was reported to play the key role in immune responses and inflammation during the development of sepsis. *Klebsiella pneumoniae* (*K. pneumoniae*) is one of the important pathogens causing sepsis. However, the function of gene *vk055_4013* which is highly homologous to *ygiM* of *E. coli* has not been demonstrated in *K. pneumoniae*. In this study, we prepared Δ*ygiM* of *K. pneumoniae* ATCC43816, and found that the deletion of *ygiM* did not affect bacterial growth and mouse mortality in the mouse infection model. Interestingly, Δ*ygiM* not only resulted in reduced bacterial resistance to macrophages, but also attenuated pathological manifestations in mouse organs. Furthermore, based on the data of Gene Expression Omnibus, the expression profiles of micro RNAs (miRNAs) and messenger RNAs (mRNAs) in the serum of 44 sepsis patients caused by *K. pneumoniae* infection were analyzed, and 11 differently expressed miRNAs and 8 DEmRNAs associated with the membrane function were found. Finally, the membrane-associated competing endogenous RNAs (ceRNAs) network was constructed. In this ceRNAs network, DEmiRNAs (hsa-miR-7108-5p, hsa-miR-6780a-5p, hsa-miR-6756-5p, hsa-miR-4433b-3p, hsa-miR-3652, hsa-miR-342-3p, hsa-miR-32-5p) and their potential downstream target DEmRNAs (VNN1, CEACAM8, PGLYRP1) were verified in the cell model infected by wild type and Δ*ygiM* of *K. pneumoniae,* respectively. Taken together, YgiM may trigger the sepsis caused by *K. pneumoniae* via membrane-associated ceRNAs. This study provided new insights into the role of YgiM in the process of *K. pneumoniae* induced sepsis.

## Introduction

Sepsis is defined as a life-threatening organ dysfunction caused by disorder host response to infection (Singer et al., 2016). In hospital mortality from sepsis has ranged from 25 to 80% over the past few decades (Angus and Wax 2001). The overall mortality rate of sepsis exceeds that of many common cancers and the delaying of appropriate antibiotic treatment for every 1 h will reduce the proportion of survival rate by about 7.6% in the shock of sepsis (Kumar et al., 2006). Due to the lack of understanding about sepsis, there is currently no effective way to mitigate the devastating effects of sepsis on the host (Seymour and Rosengart 2015). During sepsis infection, the immune response is critical for protecting the organism from infection and self-alteration. Peroxisome, a membrane-enclosed organelle associated with the lipid metabolism and ROS metabolism, has recently been recognized as a key regulator of immune function and inflammation during the development of infection, as signaling between cells and immune pathways and may influence the production of inflammatory regulators such as cytokines and antimicrobial peptides (Kumar, Kawalek, and van der Klei 2014; Deb and Nagotu 2017; Di Cara et al., 2017). In addition, peroxisomes can activate innate immunity by triggering phagocytosis, so impaired peroxidase function reduces the body’s resistance to microbial infection (Di Cara et al., 2017). It has been reported that disturbance of peroxisome biogenesis can lead to Zellweger syndrome, manifesting as severe fulminant sepsis in neonates, resulting in neonatal death (Lucaccioni et al., 2020).

MicroRNA (miRNA), a type of non-coding RNA, regulates gene expression by binding to downstream messenger RNA (mRNA) (Carthew and Sontheimer 2009). Recently, it was reported that miR-142-5p can induce an increase in the number of peroxisomes, which is the main source of intracellular ROS. So miR-142-5p can control ROS production by inducing the number of peroxisomes (Houri et al., 2020). MiRNA, as an immunomodulatory, has potentially important significance in sepsis. Funahashi *et al.* reported that in the spleen, induction of miR-146a expression can reduce multiple organ damage caused by excessive inflammation and sepsis (Funahashi et al., 2019). In the study of Chen *et al.*, miR-133a could aggravate inflammatory responses in lung, liver, and kidney by targeting SIRT1 (Chen et al., 2020). Furthermore, Liu *et al.* reported that miR-452, induced via NF-ΚB in renal tubular cells in septic, was considered as an effective prognostic predictor for early detection of acute kidney injury in sepsis patients (Liu et al., 2020). Therefore, miRNAs play important roles in sepsis, and also can affect the function of peroxisomes.

Macrophages are professional innate immune cells, and able to engulf microorganisms and trigger immune responses leading to microbial death (Pidwill et al., 2020). Macrophages play indelible roles in sepsis caused by *K. pneumoniae* infection. For instance, macrophages are highly plastic enabling small-molecule mediators to respond to various triggers, such as inflammation, infection or tissue damage (Ofek et al., 1995; Wynn, Chawla, and Pollard 2013). The early stage of sepsis lead to the over activation of macrophages, which can lead to excessive production of proinflammatory cytokines and is considered to be one of the main reasons for the high early mortality in patients with sepsis (Wang and Deng 2008). It was reported that suppressing hyperactive macrophages can effectively interfere with the occurrence and progression of sepsis caused by bacterial infections (Chen Y. F. et al., 2019). Moreover, in the process of microbial infection, peroxisomes can assist the process of phagocytic cells by activating innate immune signals, thereby promoting survival in the face of microbial challenges (Di Cara et al., 2017). Peroxisomes specialize in several metabolic tasks, including beta-oxidation of very long fatty acids, branched-chain fatty acids, and some polyunsaturated fatty acids (Van Veldhoven 2010; Islinger et al., 2018). Studies have shown that peroxisomal beta-oxidation may be involved in fine-tuning the macrophage phenotype by affecting the dynamic lipid profile during macrophage polarization (Geric et al., 2018).

YgiM, an inner membrane protein, was firstly reported in *E. coli*, and it was confirmed to be anchored to peroxisomes in yeast and mammalian cells (Maddalo et al., 2011; Lutfullahoglu-Bal et al., 2018). Through homology alignment, we found a homologous gene *vk055_4013* with 83% high similarity to *ygiM* in the genome of *K. pneumoniae* ATCC 43816. *K*. *pneumoniae* is one of the common causes of sepsis, especially in patients with low immunity (Angus and van der Poll 2013). However, the function of YgiM in *K. pneumoniae* is not clear. Based on the function of YgiM which is associated with the peroxisomes of host cells in *E. coli* and the key roles of peroxisomes in sepsis, it is suggested that YgiM may be involved in the pathogenesis of sepsis. In recent years, various types of databases and bioinformatics tools were used to study the potential roles of genes or proteins. For example, Yang *et al.* reported that the MASQC database can perform quality control of N6-methyladenine in eukaryotes and prokaryotes (Yang et al., 2020). Dai and Kong *et al.* established MTGIpick and 2SigFinder to explore how to reliably identify genomic islands from a single genome (Dai et al., 2018; Kong et al., 2020). In the study of Wang *et al.*, a recursive feature selection with random forest was developed to provide convenient for protein structural class prediction (Wang et al., 2021). Yang *et al.* constructed HPVMD-C database to provide valuable resources for HPV vaccine research and cervical cancer treatment (Yang et al., 2022). In this study, we also investigated the potential roles of YgiM in sepsis caused by *K. pneumoniae* through using the data of Gene Expression Omnibus (GEO) to construct and validate the membrane-associated ceRNAs network of host macrophages.

## Materials and methods

### Plasmids, bacterial strains, primers and growth conditions

Strains and plasmids used in this study are listed in Supplementary Table S1. Primers used in this study were from Sangon Biotech (Shanghai, China) and are listed in Supplementary Tables S2, S3. Bacterial strains were grown in lysogeny broth (LB) medium (1 g yeast extract, 1 g tryptone, 0.5 g NaCl [pH 7.2 to ∼7.4] per 100 ml deionized water). Antibiotics were added at the following concentrations: 30 μg/ml apramycin, 100 μg/ml rifampicin, 100 μg/ml ampicillin for *E. coli* or *K. pneumoniae* strains. Unless otherwise noted, all bacteria were cultured at 180 rpm in LB medium at 37°C.

### Construction of the *ygiM* deleted mutant

The *ygiM* mutant was constructed by using the CRISPR/Cas9 mediated genome-editing system (Wang et al., 2018). The pCas plasmid expresses the Cas9 protein and the λRed recombinant protein under the control of L-arabinose induction, and pSGKP plasmid expresses sgRNA. Furthermore, the pCas and pSGKP plasmids contained temperature sensitive replicon and sucrose-sensitive gene, respectively.

To generate the *ygiM* mutant, wild-type (WT) gene sequences were analyzed via an online web server (http://crispr.tefor.net/) to identify the appropriate 20-nt base-pairing regions (N20) of sgRNA. Only one of this N20 exists in the whole *K. pneumoniae* genome. In addition, another feature of the N20 sequence is that it is followed by an “NGG” sequence designed to target the guide RNA to the *ygiM.* Thereby attracting the Cas9 protein to act as molecular scissors to snip the *ygiM* in the genome. Then, the sgRNA fragment flanked by SpeI and XbaI restriction sites, along with the N20, was amplified with forward primer SpeI-Δ*ygiM*-gRNA and reverse primer XbaI-gRNA by using the pSGKP-rif plasmid as the template. The PCR product was subsequently inserted into the XbaI and SpeI digested pSGKP-rif plasmid, generating the final pSGKP plasmids with targeted sgRNA (pSGKP-YgiM-N20). Finally, the constructed pSGKP plasmid and homology arms were co-transferred into *K. pneumoniae* WT strain containing the telegenic pCas-apr plasmid to generate the *ygiM* deletion. The cultures were plated on LB agar plates containing 30 μg/ml apramycin and 100 μg/ml rifampicin, and the mutants were confirmed in selected colonies by both PCR and Sanger sequencing. The plasmids of pSGKP and pCas-apr deletion were finally depleted by incubation on LB agar medium at 37° and 5% sucrose.

### Complementation of the *ygiM* mutant

Plasmid pBAD24 was used to prepare the complementary strain of Δ*ygiM*. It was generated by PCR amplification of the *ygiM* from the chromosome of *K. pneumoniae* WT strain and subsequent cloning into EcoRI-XbaI of the pBAD24 plasmid. The recombinant plasmid pBAD24-*ygiM* was transformed into *E. coli* DH5α and then primers YgiM-F and YgiM-R were used to confirm positive colonies on LB agar with 100 μg/ml ampicillin. The *ygiM* mutant was transformed with the pBAD24-*ygiM* plasmid and named as *C-ygiM*.

### Bacterial growth curves

Growth curves of *K. pneumoniae* WT and Δ*ygiM* were determined by subculturing in LB medium and growth. These strains were shaken overnight and then added to 100 ml fresh LB at a ratio of 1:1,000 the next day, shaken at 180 rpm at 37°C. The OD_600_ of bacteria was measured once every hour, then the bacteria in each time period were diluted ten-fold and the number of colonies (CFU/ml) was calculated after overnight incubation at 37°C.

### Cell culture and *K. pneumoniae* infection *in vitro*

THP-1 human monocytes were seeded in 6-well tissue culture plates and grown in RPMI1640 containing 10% heat-inactivated fetal bovine serum (Biosharp, China) and treated with 10 ng/μl of phorbol 12-myristate 13-acetate (PMA) for 72 h to differentiate into macrophages. As for bacteria, *K. pneumoniae* was cultured to 10^8^CFU in 10 ml of LB broth. Macrophages were infected with a MOI of 50 in a final volume of 2 ml RPMI 1640 tissue culture medium supplemented with 10% heat-inactivated FBS. After 2 h, cells were washed 3 times with PBS and then incubated with RPMI1640 containing 10% fetal bovine serum and gentamicin (200 μg/ml) for 1 h to remove extracellular bacteria. Studies have shown that the internalization of *K. pneumoniae* by THP-1 cells is most obvious at 24 h (Ares et al., 2016; Maisonneuve et al., 2017; Xu et al., 2021). In this study, 40 μg/ml gentamicin was used to maintain the killing concentration of extracellular bacteria to 24 h, and cells were lysed with 0.1% Triton X-100. Then, 10-fold serial dilutions were plated on LB agar plates and total CFU was determined. At the same time, to confirm that *K. pneumoniae* could be phagocytosed by macrophages, we also constructed a *K. pneumoniae* strain containing green fluorescent protein. Briefly, GFP^+^ plasmid was provided by Rao from Third Military Medical University (Rao et al., 2022). This GFP^+^ plasmid was introduced into *K. pneumoniae* ATCC43816. The macrophages were fixed using 4% formaldehyde and 0.1% Triton, permeabilized and stained with phalloidin-Alexa 594 (Beyotime, China) and DAPI (Beyotime, China) to visualize the actin filaments and the nuclei acid, respectively. The labelled cells were observed by fluorescence observation (Zeiss, Germany).

### Mouse infection assay

To investigate whether *ygiM* affects bacterial virulence, bacteria were injected into the bloodstream of mouse model via tail vein injection. 6–8 weeks C57BL/6 mice were used with 10 mice in each group, and 10^6^ CFU bacteria was injected into the tail vein, observed for 5 days, and the corresponding survival curve was calculated. The bacteria were shaken overnight, diluted at 1:100 the next day, cultured to 10^8^ CFU/ml, serially diluted 10 times with phosphate buffered saline (PBS), and inoculated on LB agar with appropriate dilution. On the plate, the corresponding CFU number was calculated. In addition, C57BL/6 mice were divided into three groups, each group contained three mice, 48 h after the injection of 10^6^ CFU colonies through the tail vein, the liver and lung tissues of the mice in each group were taken, embedded, stained with hematoxylin-eosin, and the tissues were obtained. Pathological examination to understand the pathological changes. All animal experiments were established by the Soochow University Animal Care and Utilization Committee and complied with all ethics and animal husbandry regulations.

### Identification of DEmiRNAs and DEmRNAs by microarray data

The microarray data GSE174507 and GSE13904 were obtained from GEO (https://www.ncbi.nlm.nih.gov/geo/) which is an online public gene data repository for research. In total, the miRNA expression data obtained from GSE174507 included 12 peripheral blood samples from sepsis patients and six peripheral blood samples from control donors. The mRNA raw data obtained from GSE13904 included 32 whole blood samples from sepsis patients adopted at the first day and 18 whole blood samples from healthy individuals. The above raw microarray data was extracted from the GEO database. So, this study was not required any ethical review and informed consent because of the public availability of GEO data.

The DEmiRNAs and DEmRNAs were identified by the expression difference between the sepsis and healthy individuals’ samples of the microarray data. The *p*-value and the absolute log value of fold-change (log|FC|) were analysis in R language by the limma package. The log|FC|> 1.0 and *p*-value <0.05 were the selection criteria to define the differentially expressed genes.

### Transcription factors and pathway enrichment analysis

Transcription factors, as the direct downstream targets of miRNA, are essential molecules in immunity to bacterial infection. Based on the DEmiRNAs identified, FunRich (Version 3.1.3) was used to analyze and visualize the differential expression transcription factors. FunRich is a stand-alone software tool used mainly for functional enrichment and interaction network analysis of genes and proteins.

Further, we used Gene Ontology (GO) annotation (http://www.geneontology.org) and Kyoto Encyclopedia of Genes and Genomes (KEGG) pathway to determine the functions of the DEmRNAs. The clusterProfler package in R language was employed.

### Construction of the membrane-related interaction network

In the results of the differential analysis of the high-throughput sequencing data information, the DEmiRNAs target genes were predicted by databases of miRDB, miRTarBase and TargetScan. In this study, we use the prediction of DEmiRNAs target genes to intersect with identified differentially expressed downstream mRNA to further screen the prediction results. And we used the prediction results to construct a ceRNA interaction network.

The exploration of protein interactions helps reveal the underlying pathological mechanism in children’s sepsis. In this study, we used the Search Tool for the Retrieval of Interacting Genes/Proteins (STRING) database (https://string-db.org/) to construct a PPI network. The clustered sub-networks and hub genes were identified by employing plugins of Cytoscape, Molecular Complex Detection (MCODE), and cytoHubba (version 3.8.0).

Membrane related genes were obtained from the results of GO annotation. The mRNAs annotated in membrane-related pathways were considered candidate mRNAs in the interaction subnetwork. Upstream miRNAs were predicted using the miRDB, miRTarBase, and TargetScan databases. The candidate miRNAs in the membrane-related interaction network were the intersecting mRNAs between the prediction results and the identified DEmiRNAs. The membrane-related interaction network was constructed based on the above results and the protein-protein interaction relationships between DEmRNAs. The results were visualized using Cytoscape (version 3.8.0).

### Verification of DEmiRNAs and DEmRNAs expression

Specific validating primers for several differentially expressed RNAs (DERNAs) were designed based on the sequence of linear transcripts. Total RNA was extracted using TRIzol (Invitrogen United States) following the manufacturer’s instructions. The DERNAs complementary DNAs (cDNAs) were synthesized using miScript II RT Kit (QIAGEN, United States). The RT-qPCR was performed using SYBR Green qPCR Master Mix (QIAGEN, United States). The expression level of DEmiRNAs and DEmRNAs were normalized to the endogenous control of human glyceraldehyde-3-phosphate dehydrogenase (GAPDH) as the endogenous control. And the expression level of DEmiRNAs was normalized to the endogenous control of U6.

### Reverse transcription quantitative polymerase chain reaction

The primers were designed and synthesized by Sangon Biotech (Shanghai, China). RNA template, Primer Mix, dNTP Mix, DTT, RT buffer, HiFi-MMLV, and RNA-free water were dissolved on ice. The reverse transcription system consisted of a volume of 20 μl. The reaction solution was used for fluorescence quantitative PCR according to the instructions of the SYBR^®^ Premix Ex TaqTM II kit (Action-award Biotechnology, China). The 2^–ΔΔCt^ method was used to express the expression relationships of multiple genes between the experimental group and control group. All of the qRT-PCR assays were repeated at least three times.

### Statistical analysis

Sepsis children and healthy individuals were compared to evaluate the statistical significance between the two groups. All data analysis was performed using R software (version 4.0.1), FunRich (version 3.1.3), Cytoscape (version 3.8.0) and GraphPad Prism 8. The *p* value of <0.05 was considered statistically significant.

## Results

### Two plasmid system pCas-pSGKP for *ygiM* deleting

**FIGURE 1:**
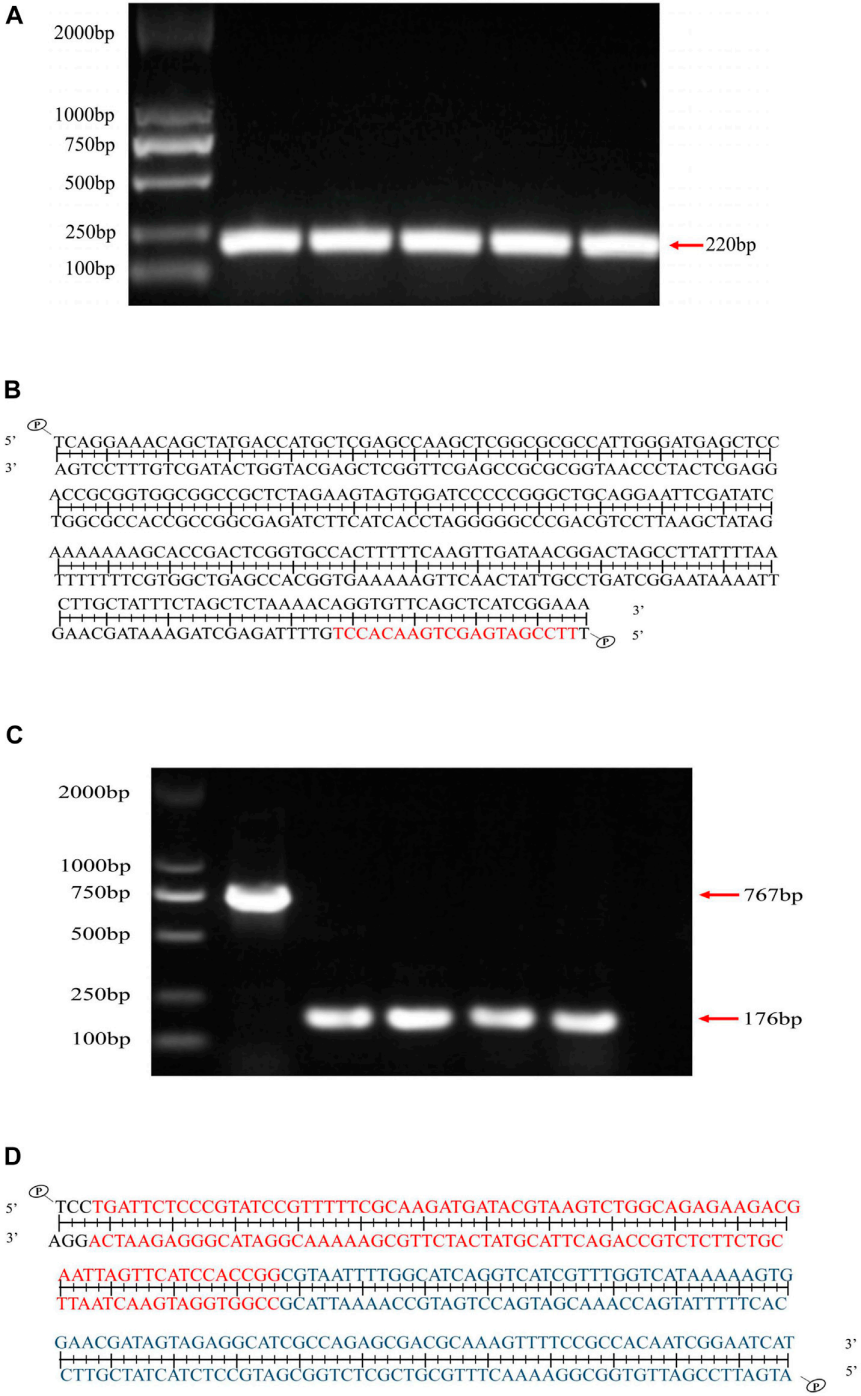
Verification of *ygiM* deletion mutant by PCR and sequencing. **(A)** The pSGKP plasmid contains YgiM protein N20, and the target band is 220bp after PCR verification. **(B)** Sanger sequencing indicated that the pSGKP plasmid containing YgiM protein N20 was successfully constructed (red bases represent N20). **(C)** PCR confirmed that the *ygiM* mutant was constructed successfully. **(D)** Sanger sequencing verified that *ygiM* was successfully knocked out. (Red bases represent upstream of *ygiM* and blue bases represent downstream of *ygiM*).

The pSGKP plasmid containing *ygiM*-N20 was confirmed by PCR (Figure 1A), and subsequently the PCR products were confirmed by DNA sequencing (Figure 1B). In addition, the pSGKP plasmid carried the rifampicin gene for screening. As shown in Figure 1C, the *ygiM* mutant was verified by PCR. The result of DNA sequencing showed that *ygiM* was successfully knocked out (Figure 1D).

### Deletion of *ygiM* did not affect the bacterial growth

**FIGURE 2:**
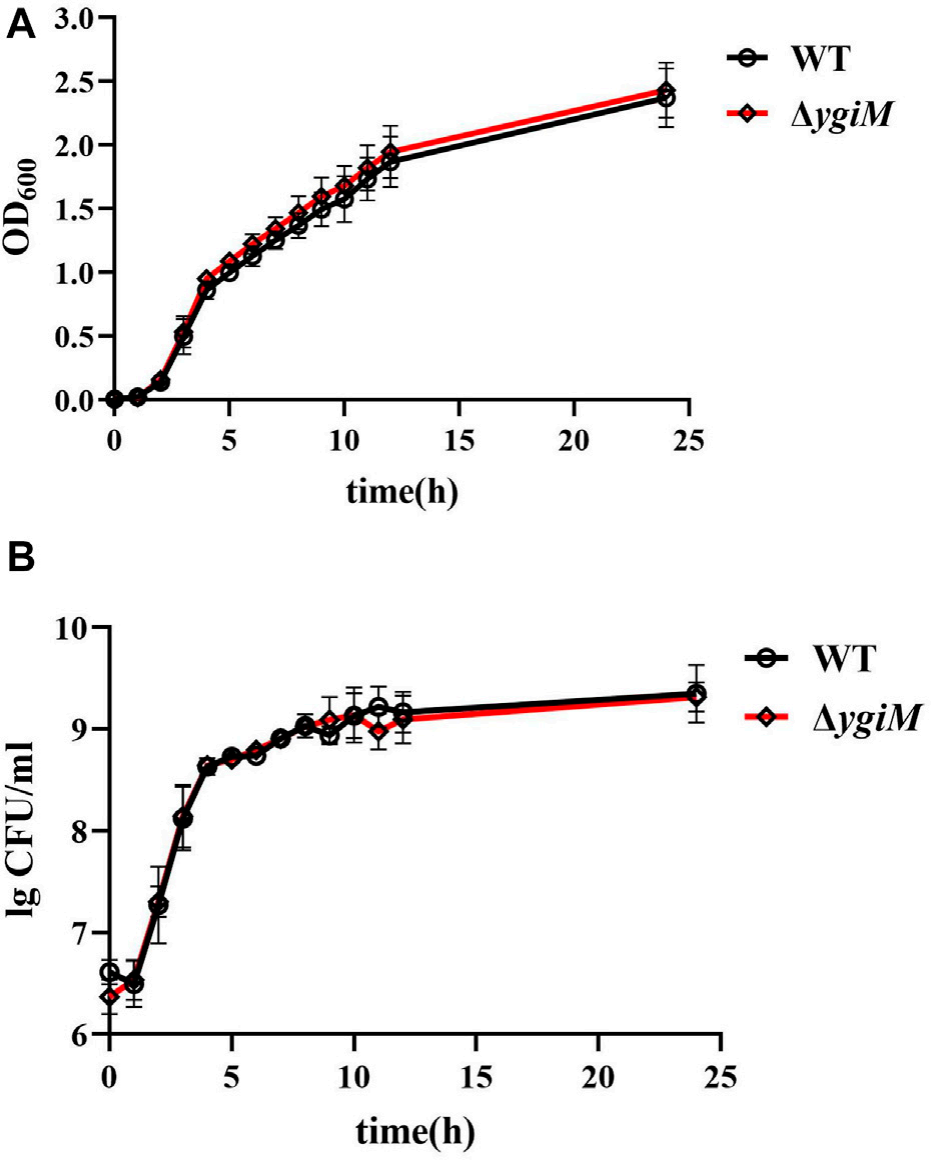
Growth curve and bacterial colony number of *K. pneumoniae* wild-type strain and *ygiM* mutant strains. **(A)** Comparison the growth curves of wild-type and *ygiM* mutant strains. The experiments were independently repeated three times. **(B)** Comparison the CFU/ml of wild-type and *ygiM* mutant strains. The experiments were independently repeated three times.

To investigate whether *ygiM* affects the growth or colony size of *K. pneumoniae*, we measured the OD_600_ and specific CFU/ml of WT and Δ*ygiM* strains every hour. The growth curves of these two strains in LB broth for 24 h were similar (Figure 2A). The results showed that the colony morphology of the two strains on LB agar plates was also similar (Figure 2B).

### YgiM contributes to bacterial resist against phagocytosis by macrophages

**FIGURE 3:**
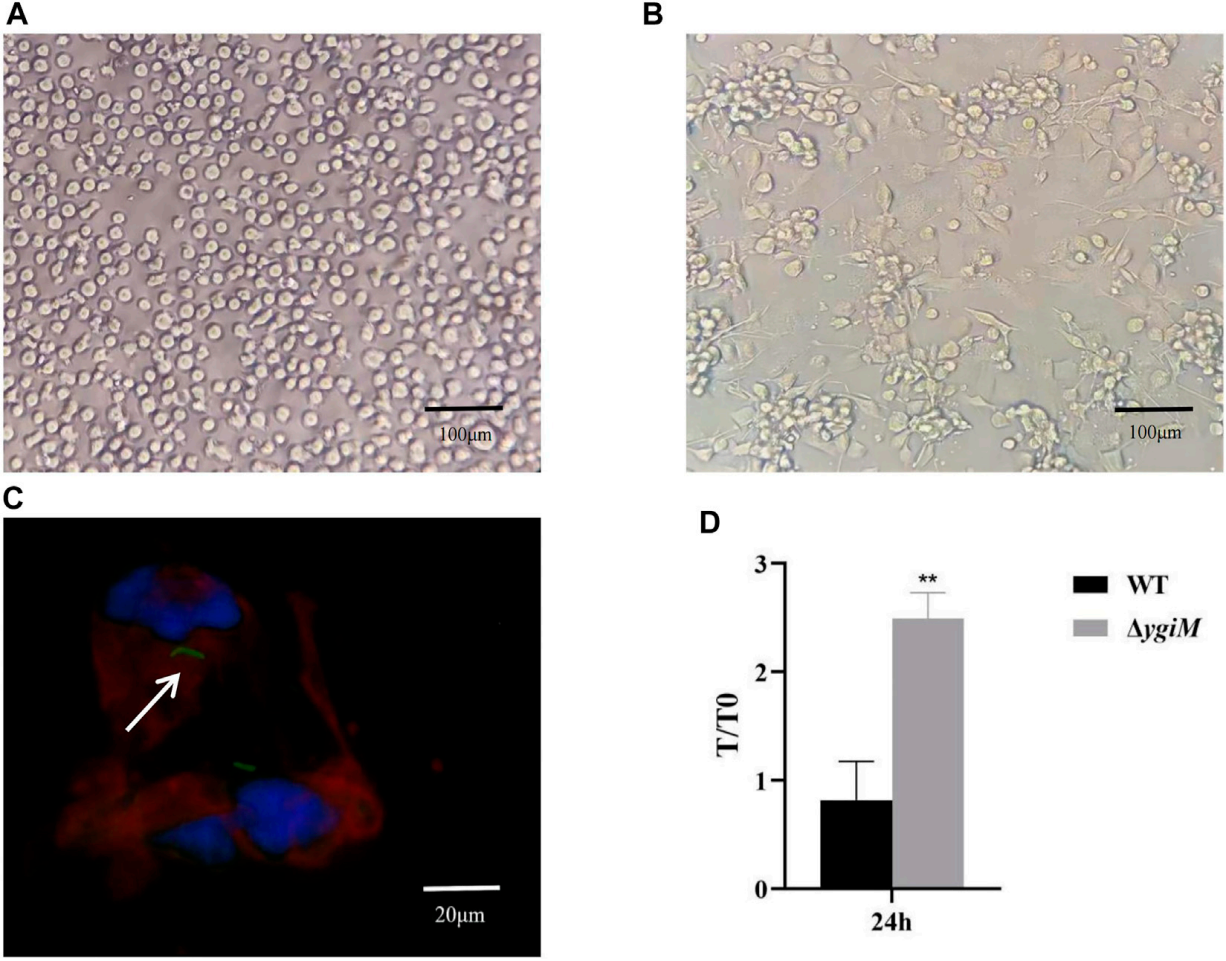
Determination of phagocytosis resistance of the wild type and the *ygiM* gene mutant strains. **(A)** THP-1 cells before induction (round, clear nuclei). **(B)** THP-1 cells are induced into macrophages by PMA (with pseudopodia, indistinct nuclei). **(C)** Fluorescence images of macrophages phagocytosing *K. pneumoniae.* The arrow indicates fluorescence labelled intracellular *K. pneumoniae.* **(D)** Plate to count macrophage phagocytosis. Data is displayed as intracellular bacterial counts of macrophages from three independent experiments (**: *p* < 0.01).

To assess whether *ygiM* affects pathogenic processes caused by increased phagocytic sensitivity of macrophages, we investigated the antiphagocytic ability of WT and Δ*ygiM* strains. As shown in Figure 3A, THP-1 cells were round with clear nuclei before induction by PMA. After 72 h induction by PMA, the protruding pseudopodia became irregular macrophages with blurred nuclei (Figure 3B). In the fluorescence experiment, we saw that the THP-1-derived macrophages phagocytosed *K. pneumoniae* (Figure 3C)*.* Our results showed that more Δ*ygiM* strains were recovered from THP-1-derived macrophages than WT at 24 h co-culture of cells and bacteria. It was suggested that *ygiM* may enhance the bacterial antiphagocytic ability to macrophages (Figure 3D).

### The potential roles of *ygiM* of *K. pneumoniae* in the formation of liver abscesses and pneumonia

**FIGURE 4:**
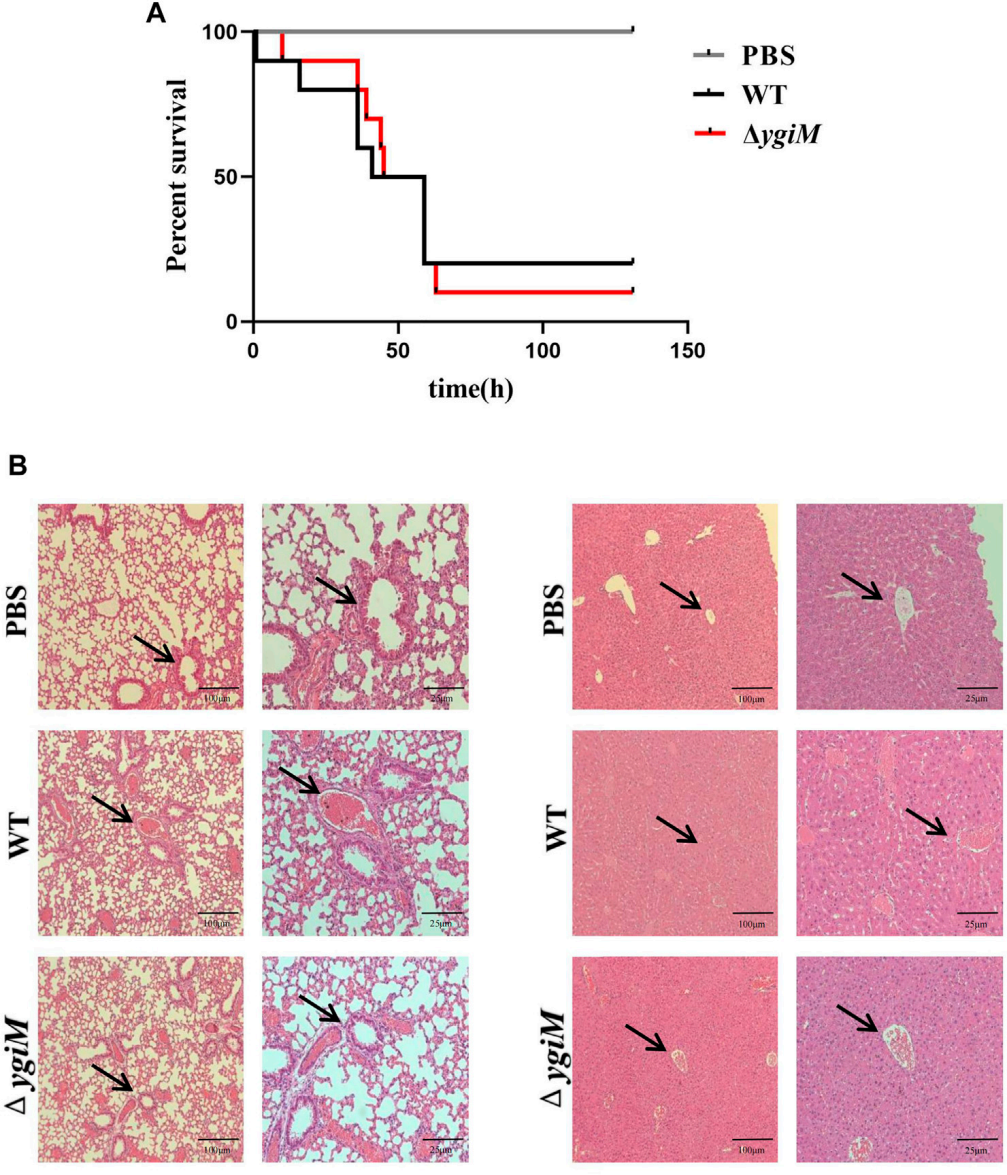
Survival curves and pathological morphology of mice. **(A)** Survival curves of mice in the tail vein infection model. Ten mice each of PBS, WT and Δ*ygiM* were injected with 10^6^ CFU through the tail vein, respectively. The 5 days survival rate of mice was determined. **(B)** Histopathology of mouse lung and liver after infection with PBS (negative control), WT and Δ*ygiM*. 48 h after infection, the lungs and livers of different groups of mice were dissected and stained. These numbers represent at least three sections from three mice that exhibited similar pathology in their tissues. The arrows indicate the obvious pathological differences.

To determine whether *ygiM* is involved in the virulence of *K. pneumoniae*, C57BL/6 mice were infected with 10^6^ CFU of bacteria by tail vein injection. The number of dead mice was counted for five consecutive days (Figure 4A). The results showed that the survival rate of group infected with WT strains was 20% after infection for 72 h, and that of the group infected with Δ*ygiM* was 10%. So there was no significant difference in the overall survival rate curve between these two groups. We then compared the pathological morphology of the liver and lung of mice infected with PBS, WT and Δ*ygiM* groups. Compared with PBS treated mice, The mice infected by WT strain had damaged lung tissue and severe liver lobule congestion. However, the degree of lung tissue damage and hepatic lobular congestion in mice treated with Δ*ygiM* strain was alleviated (Figure 4B).

### Identification of DEmiRNAs and DEmRNAs

The log|FC| > 1.0 and *p* < 0.05 were the selection criteria to define differentially expressed genes. In the miRNA dataset, 21 upregulated miRNAs and 17 downregulated miRNAs were identified (Supplementary Figures S1A,B). In the mRNA dataset, 55 upregulated mRNAs and four downregulated mRNA were identified (Supplementary Figures S2A,B).

### Transcription factors and pathway enrichment analysis

**FIGURE 5:**
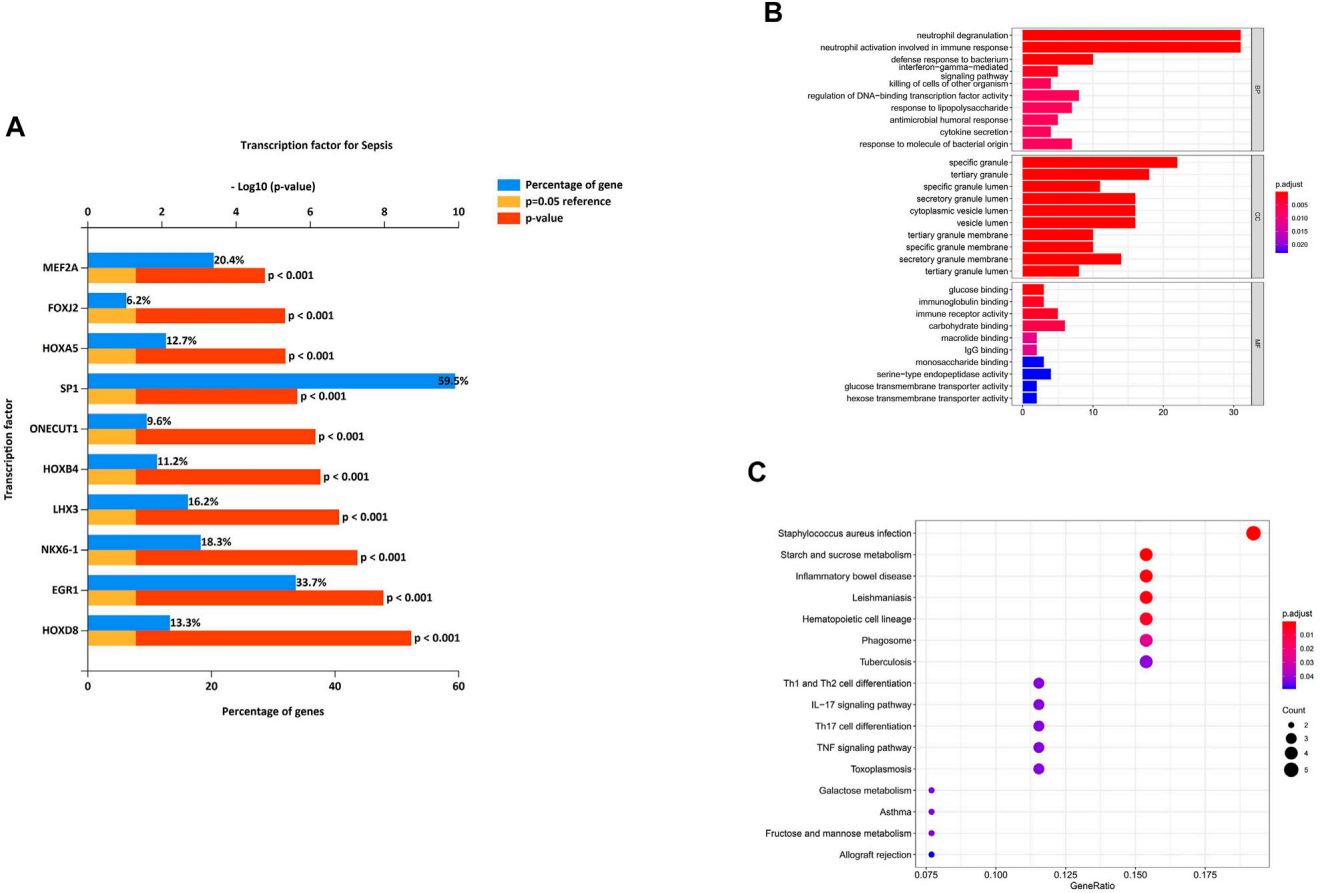
Characteristics and functional analysis of differentially expressed genes (DEGs). **(A)** Differentially expressed transcription factors associated with the identified DEmiRNAs. HOXD8, EGR1, NKX6-1, LHX3, HOXB4, ONECUT1, SP1, HOXA5, FOXJ2 and MEF2A were the most significant molecules in sepsis. **(B)** GO enrichment analysis of the DEmRNAs in biological process (BP), cellular component (CC) and molecular function (MF) subgroups. **(C)** KEGG enrichment analysis of DEmRNAs.

We analyzed the differential expression of transcription factors by the 38 identified DEmiRNAs and identified 50 statistically significant transcription factors. HOXD8, EGR1, NKX6-1, LHX3, HOXB4, ONECUT1, SP1, HOXA5, FOXJ2 and MEF2A were the most significant molecules in sepsis (Figure 5A). Among these, HOXD8 was the most statistically significant transcription factor. And SP-1 was the transcription factor with the highest percentage.

To better understand the potential functions of the identified DEmRNAs the initiation of sepsis, GO annotation and KEGG pathway analyses were performed. In GO annotation analysis, we found that the DEmRNAs were significantly enriched in the terms of neutrophil degranulation, neutrophil activation involved in immune response, and defense response to bacterium in the BP subgroup. Specific granule, tertiary granule, and specific granule lumen were the most significant GO terms in the CC subgroup. The top three GO processes included glucose binding, immunoglobulin binding, and immune receptor activity in the MF subgroup for DEmRNAs (Figure 5B).

In KEGG pathway enrichment analysis, starch and sucrose metabolism and inflammatory bowel disease were the most significant pathways enriched for DEmRNAs in the initiation of sepsis (Figure 5C).

### Construction of the membrane-related interaction network

**FIGURE 6:**
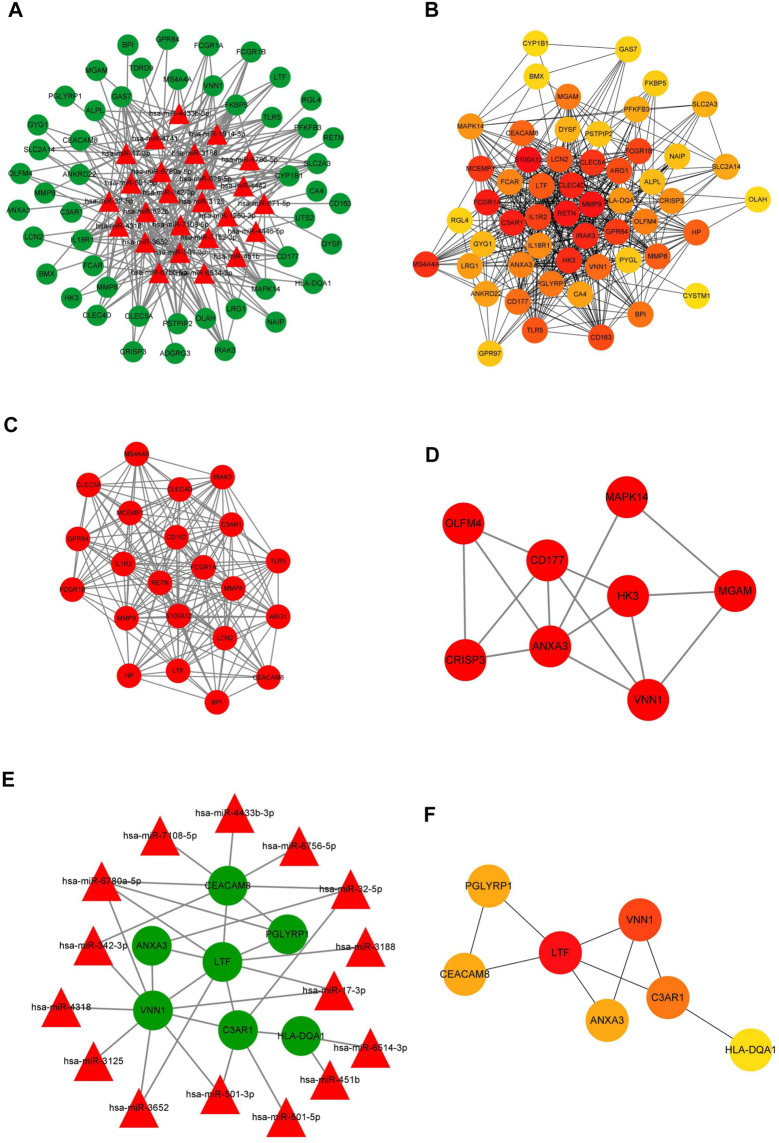
Construction of the membrane-related interaction network. **(A)** The competitive endogenous RNA (ceRNA) network of DEGs in the initiation of sepsis. In total, there were 25 miRNAs and 50 mRNAs in the ceRNA network. **(B)** The target genes of the identified DEmRNAs were ranked in the PPI network. The depth of red indicates the importance of genes in the network. **(C)** The most closely-clustered subnetwork was composed of 22 nodes and 178 edges. **(D)** The clustered subnetwork identified by the MCODE plug-in in Cytoscape had 8 nodes and 15 edges. **(E)** The membrane-related interaction network in the initiation of sepsis. Based on the results of the prediction of DEmiRNAs and PPI network, we constructed a autophagy interaction network including 15 miRNAs and seven mRNAs. **(F)** In the membrane-related interaction network, LTF, VNN1, and C3AR1 were the most important hub gene among the identified genes. The depth of red indicates the importance of genes in the network among mRNAs.

To better understand the endogenous regulatory mechanism of differentially expressed genes (DEGs) in the initiation of sepsis, we predicted the downstream target genes of DEmiRNAs by employing the miRDB, miRTarBase, and TargetScan databases. At the miRNA level, 25 miRNAs were predicted, and following a comparison with DEmRNAs, we confirmed 50 intersecting mRNAs in the ceRNA network (Figure 6A).

Further, we used the identified DEmRNAs to construct a PPI network, which included 54 nodes and 912 edges, under the condition that unconnected points were removed. Among the 54 genes, 40 genes had a score >100 analyzed by the maximal clique centrality (MCC) method in Cytohubba (Figure 6B). The top five hub genes were S100A12, MMP9, RETN, FCGR1A, and C3AR1. We also defined the most closely clustered sub-network by employing the MCODE plug-in in Cytoscape, which consisted of 22 nodes and 178 edges in which FCGR1A, S100A12, RETN were the most important genes (Figure 6C). In addition, we identified other clustered sub-network, with 8 nodes and 15 edges in which ANXA3, CD177, and VNN1 were considered as hub genes in this network (Figure 6D).

Due to the potential interesting roles of *ygiM* in sepsis, a membrane-related interaction network was constructed based on analysis of the ceRNA and PPI networks. Membrane-related genes were obtained from the results of GO annotation. Seven differentially expressed membrane-related genes were identified in which upstream target genes of these membrane-related genes were identified in sepsis patients at the initial stage. A total of 15 miRNAs and seven mRNAs were involved in network construction (Figure 6E). In the membrane-related interaction network, LTF, VNN1, and C3AR1 were the most important hub gene among the identified genes (Figure 6F).

### Verification of autophagy-related genes by using THP-1-derived macrophages

**FIGURE 7:**
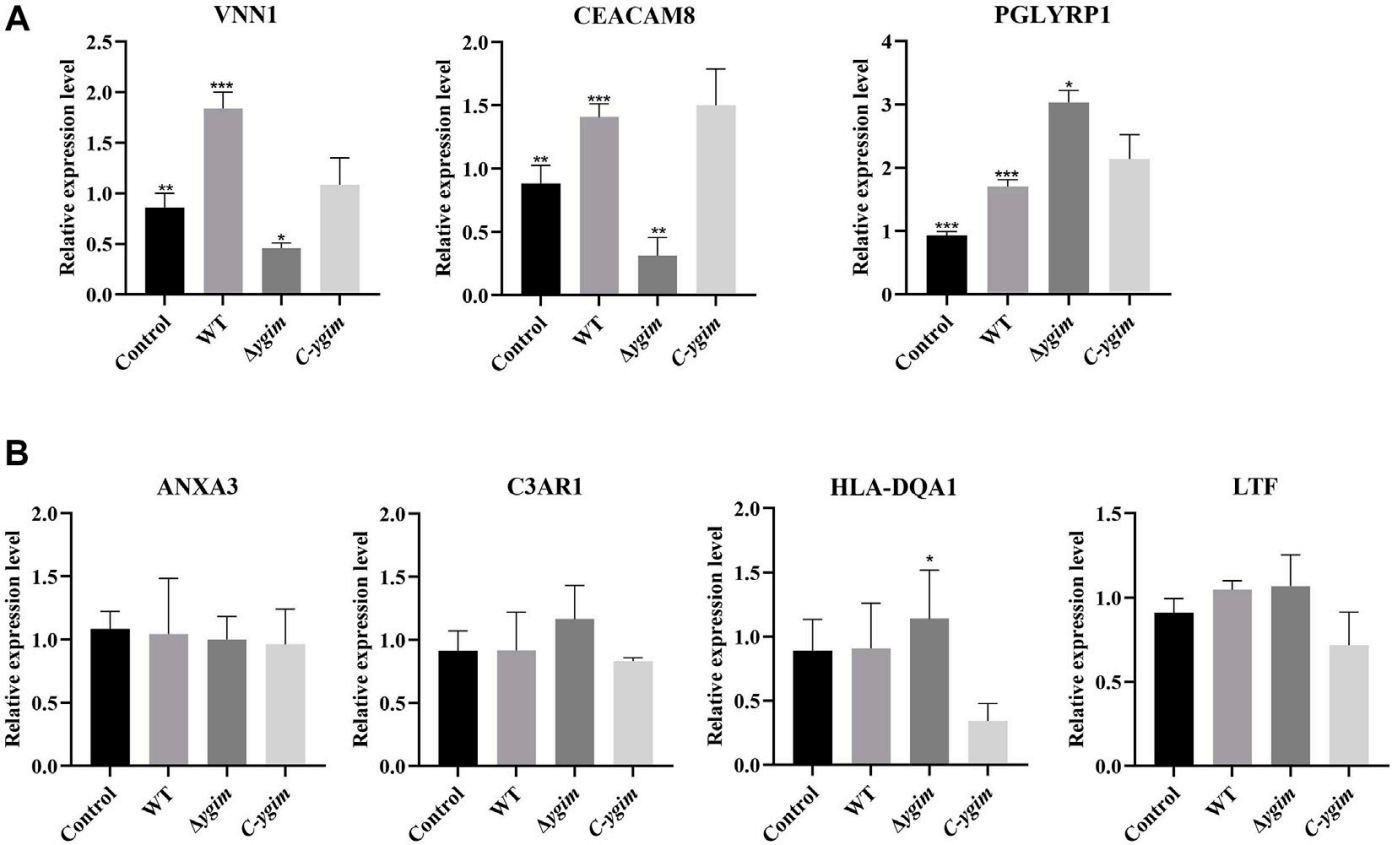
The relative expression of mRNA in *K. pneumoniae* wild-type, Δ*ygiM* and *C-ygiM* strains. **(A)** Positive targets of mRNA: VNN1, CEACAM8, PGLYRP1 were considered to be statistically significant in the infection of *K. pneumoniae* control, wild type and Δ*ygiM* groups (*: *p* < 0.05, **: *p* < 0.01, ***: *p* < 0.001). **(B)** Negative target of mRNA: C3AR1, HLA-DQA1, ANXA3, LTF were considered to be statistically insignificant in the infection of *K. pneumoniae* control, wild type and Δ*ygiM* groups (*: *p* < 0.05).

According to the above search conditions, seven potential targets mRNAs of the above DEmiRNAs were found. Of which, three target mRNAs (VNN1, CEACAM8, and PGLYRP1) were shown to be associated with the *ygiM*. As shown in Figure 7A, compared with the WT group, VNN1 and CEACAM8 in Δy*giM* group showed significant downregulation. However, the Δy*giM* group of PGLYRP1 showed significant upregulation compared to the wild group. In addition, C3AR1, HLA-DQA1, ANXA3 and LTF had no differential expression in the comparison of the control and WT, the WT and Δ*ygiM* group, Δ*ygiM* and *C-ygiM* group respectively (Figure 7B).

**FIGURE 8:**
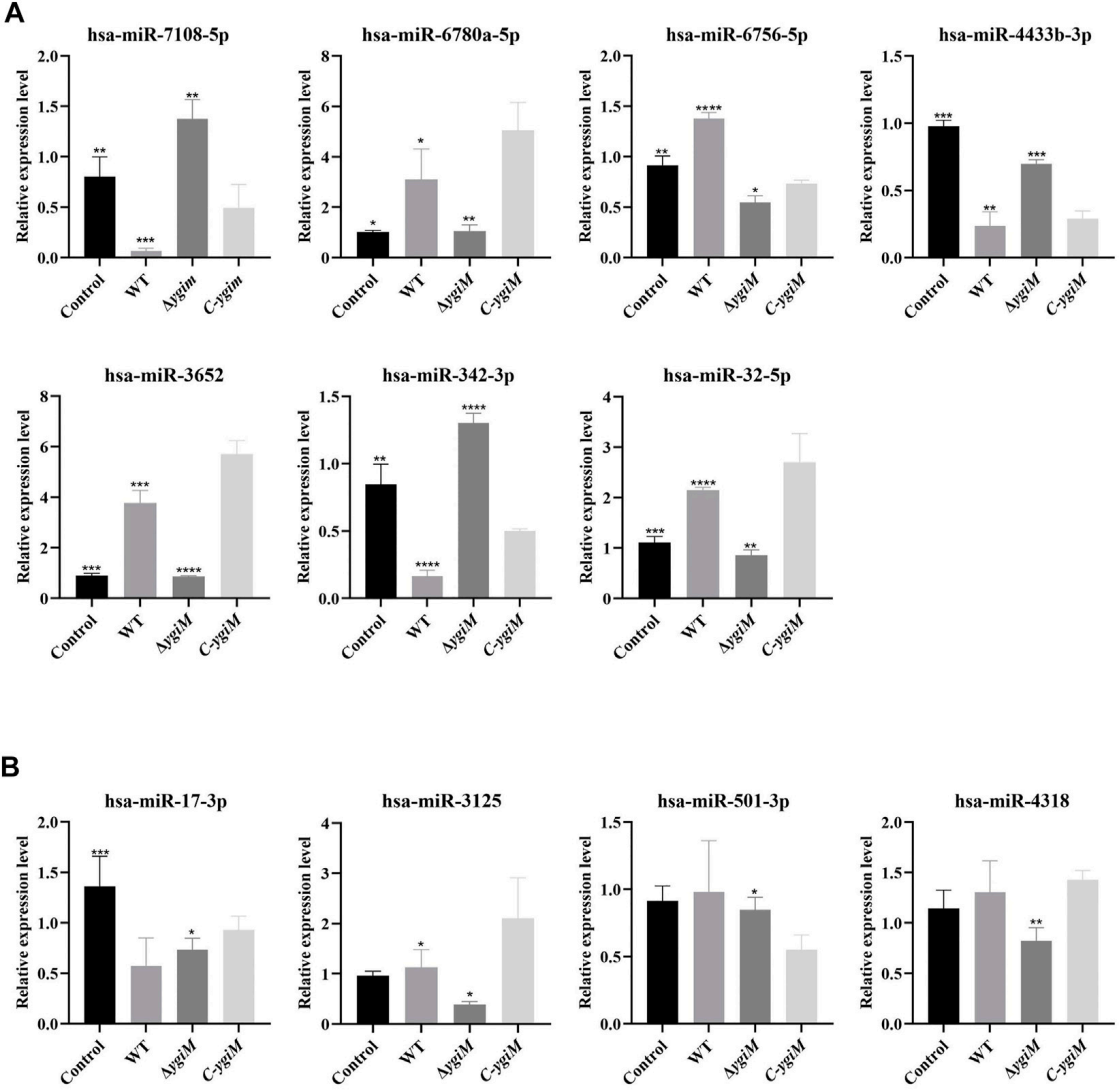
The relative expression of miRNA in *K. pneumoniae* wild-type, Δ*ygiM* and *C-ygiM* strains. **(A)** Positive targets of miRNA: hsa-miR-7108-5p, hsa-miR-6780a-5p, hsa-miR-6756-5p, hsa-miR-4433b-3p, hsa-miR-3652, hsa-miR-342-3p and hsa-miR-32-5p were considered to be statistically significant in the infection of *K. pneumoniae* control, wild type and Δ*ygiM* groups (*: *p* < 0.05, **: *p* < 0.01, ***, *p* < 0.001: ****: *p* < 0.0001). **(B)** Negative target of micRNA: hsa-miR-501-3p, hsa-miR-17-3p, hsa-miR-3125 and hsa-miR-4318 were considered to be statistically insignificant in the infection of *K. pneumoniae* control, wild type and Δ*ygiM* groups (*: *p* < 0.05, **: *p* < 0.01, ***: *p* < 0.001).

Based on the results, the miRNAs with differentially expressed downstream mRNAs were further explored. Differential expression of seven miRNAs (hsa-miR-7108-5p, hsa-miR-6780a-5p, hsa-miR-6756-5p, hsa-miR-4433b-3p, hsa-miR-3652, hsa-miR-342-3p, hsa-miR-32-5p) were considered to be statistically significant in the infection of *K. pneumoniae* WT and Δ*ygiM* (Figure 8A)*.* In addition, hsa-miR-501-3p, hsa-miR-17-3p, hsa-miR-3125 and hsa-miR-4318 had little differential expression in the comparison of the control, WT and Δ*ygiM* mutant groups (Figure 8B).

In all DEmiRNAs and DEmRNAs, the target expression trend of the *C-ygiM* group was close to that of the WT group. After comparing the results of miRNAs and mRNAs, we identified hsa-miR-342-3p/VNN1, hsa-miR-7108-5p/CEACAM8, hsa-miR-4433b-3p/CEACAM8, and hsa-miR-342-3p/CEACAM8 as interactive pairs related *ygiM* that were statistically significant in sepsis.

## Discussion

Sepsis can have serious consequences for the host. And can kill 20 to 50 percent of people with severe infections (Felbinger, Suchner, and Goetz 1999; Cohen et al., 2015). Peroxisomes play important roles in the occurrence and development of sepsis. Peroxisomes are multifunctional organelles that rely on reliable substrate and cofactor transport systems to complete various biological processes. Such as fatty acid oxidation (FAO) and hydrogen peroxide (H_2_O_2_) metabolism (Platta and Erdmann 2007; Vasko 2016). Also, sepsis leads to disruption of fatty acid oxidation, which is reflected in the downregulation of peroxisome proliferator-activated receptor alpha (PPARα) (Han et al., 2020). Thus peroxisome dysfunction leads to oxidative stress, which interferes with the pathological changes of sepsis. Studies have shown that *K. pneumoniae* is the major pathogen causing localized infections such as pneumonia and liver abscesses, and disseminated infections that can lead to severe sepsis (Chew, Lin, and Teo 2017). In *K. pneumoniae*, the inner membrane protein YgiM has been shown to play an important role by localizing peroxisomes in yeast and mammalian cells (Lutfullahoglu-Bal et al., 2018). The present study explored whether YgiM protein deficiency affects the process of *K. pneumoniae* interfering with the host, and revealed a membrane associated ceRNAs network of YgiM protein in *K. pneumoniae* induced sepsis. Most importantly, this study has opened up some new targets to a certain extent, which can be used as a reference for the treatment of sepsis.

Since *K. pneumoniae* has capsule, its resistance to macrophage phagocytosis is one of its virulence mechanisms. The presence of the capsule can act as a protective shield for bacterial antimicrobial peptides, inhibiting the early inflammatory response (Cortes et al., 2002; Al-Awad et al., 2020). Studies have shown that peroxisomes are required for the phagocytosis of bacteria by macrophages and that reduced peroxisome function impairs the response to bacterial challenges (Di Cara et al., 2017). Our results show that Δ*ygiM* results in an enhanced ability of cells to phagocytose bacteria, and thus an enhanced ability of cells to clear bacteria. This further suggests that *ygiM* can attenuate the host’s attack against *K. pneumoniae* by targeting the host’s peroxisomes, thereby making it easier for the bacteria to survive and become pathogenic in the host, whereas Δ*ygiM* makes it impossible for the bacteria to target the host’s peroxisomes, thereby increasing the ability of macrophages to clear bacteria. To further test this hypothesis, we established a mouse bloodstream infection model to explore whether the loss of *ygiM* affects the pathogenicity of bacteria to the host. The results showed that though Δ*ygiM* did not affect the virulence of *K. pneumoniae*, attenuated congestion and inflammation in the liver and lungs of mice, suggesting that the presence of *ygiM* enhanced the pathogenicity of *K. pneumoniae*, but not virulence affecting the entire genome.

Several mRNA targets in sepsis were identified in this study. Based on the results of GO annotation, membrane-related genes were obtained. Further, the membrane-related interaction network was proposed from ceRNA network by the analysis of the miRDB, miRTarBase, and TargetScan databases. It is worth mentioning that eight mRNAs were emerged in the GO annotation in total. Because of the upstream miRNAs of HLA-DRB4 were not annotated, it was not shown in the membrane-related interaction network. But the interesting differential expression of HLA-DRB4 was revealed with *ygiM* (data show in Supplementary Figure S3). The role of HLA-DRB4 in sepsis also deserves further study in the future. Among the mRNAs related *ygiM* we verified, VNN1, CEACAM8, and PGLYRP1 were highlighted about sepsis firstly. Among the four mRNAs, VNN1 is an important PPARα target gene, and the production of VNN1 depends on PPARα activity (Rommelaere et al., 2013). PPARα (peroxisome proliferator-activated receptor α) is a ligand-activated transcription factor that plays a major role in different aspects of hepatic lipid metabolism (Kliewer et al., 1997). PPARα expression and activation protects the body from sepsis by promoting an appropriate metabolic response (Paumelle et al., 2019). Our results showed that VNN1 increased after *K. pneumoniae* infection compared with the control group, but VNN1 decreased to a certain extent due to *ygiM* deficient, suggesting that it may play a pro-inflammatory role in *K. pneumoniae* infection. CEACAM8, also known as plasma membrane carcinoembryonic antigen-related cell adhesion molecule, is only expressed in cells of the granulocyte lineage, especially neutrophils (Zhao et al., 2004; Ribon et al., 2019). Neutrophils are not only typical pro-inflammatory cells, but also have immunomodulatory properties, so CEACAM8 may be involved in innate immunity in sepsis (Ribon et al., 2019). Our results showed that WT CEACAM8 was elevated compared with controls, suggesting that *K. pneumoniae* can promote neutrophil degranulation, thereby promoting the occurrence and development of inflammation. However, the expression of CEACAM8 in the *ygiM* mutant was significantly decreased, even lower than that in the control group. This further suggests that deletion of the *ygiM* reduces the inflammatory response induced by *K. pneumoniae.* PGLYRPs (peptidoglycan recognition proteins) are members of the innate immune system (Yoshida, Kinoshita, and Ashida 1996). As one of the members, PGLYRP1 is mainly present in neutrophil granules, which can be highly bound to the bacterial cell wall, thereby activating the bacterial stress defense response, resulting in bacteria dying (Dziarski et al., 2003; Dziarski, Kashyap, and Gupta 2012). Compared with the *K. pneumoniae* WT, *ygiM* resulted in increased expression of PGLYRP1, suggesting that deletion of *ygiM* enhanced resistance to *K. pneumoniae*. It may indicate that this protein may provide ideas for the development of new antibacterial agents.

Furthermore, our study noticed that transcription factors play an important role in sepsis. Chen *et al.* found that EGR1 intervention *in vivo* reduced host proinflammatory cytokine secretion and rescued survival and tissue damage in a mouse sepsis model (Chen F. et al., 2019). Wu *et al.* reported that SP1 could be considered a target of miRNA-124-3p to alleviate myocardial injury in sepsis (Wu et al., 2022). And Zhang *et al.* found that the lncRNA MIR155HG regulates MEF2A to affect apoptosis and inflammation by sponging miR-194-5p in sepsis (Zhang et al., 2021). The above-mentioned molecules were all shown in our transcription factor analysis. And the potential of the results in our transcription factor analysis was highlighted. Studies on other transcription factors in the results deserve further research in the future. Among the miRNAs related *ygiM* we verified, hsa-miR-7108-5p, hsa-miR-6780a-5p, hsa-miR-6756-5p, hsa-miR-4433b-3p hsa-miR-3652, hsa-miR-32-5p, hsa-miR-342-3p were highlighted about sepsis firstly. As for hsa-miR-342-3p, Fu *et al.* reported that miR-342-3p regulates the immunity of *Mycobacterium tuberculosis* by increasing the production of inflammatory cytokines and chemokines (Fu et al., 2021). And our study deepened the understanding of miR-342-3p in infection. MiR-342-3p may have a wide range of abilities to regulate host immune function in intracellular bacterial infections. And *ygiM* could be considered a key bacterial target that activates host immune targets. In this study, hsa-miR-17-3p, hsa-miR-3652, and hsa-miR-32-5p were verified as ideal targets for the diagnosis of sepsis in the early stage. And the potential of these targets has also attracted the interest of other researchers. Tang *et al.* reported that miR-3652 is involved in immune regulation in periodontitis (Tang et al., 2021). The study by Li *et al.* found that miR-3652 was the shared genetic and epigenetic expression target between periodontitis and oral squamous cell carcinoma (Li et al., 2018). As for miRNA-32-5p, Zhang *et al.* reported that miRNA-32-5p regulates the survival and inflammatory response of mycobacteria in *mycobacterium* tuberculosis-infected macrophages by targeting FSTL1 (Zhang et al., 2017). Feng *et al.* reported that miR-32-5p is involved in intestinal epithelial cell apoptosis induced by the activation of transforming growth factor-β-activated kinase 1 (TAK1)-p38 in *Helicobacter pylori* infection (Feng et al., 2019).

In conclusion, we report the function and role of a novel gene *ygiM* in *K. pneumoniae* and construct a network of ceRNAs of *ygiM*. Several potential novel biomarkers were discovered to understand the pathogenesis of infection by *ygiM* mutant and WT strains. Furthermore, this study supports that hsa-miR-342-3p/VNN1, hsa-miR-7108-5p/CEACAM8, hsa-miR-4433b-3p/CEACAM8 and hsa-miR-342-3p/CEACAM8 as *ygiM*-specific interaction networks. Although *ygiM* did not affect the overall virulence of *K. pneumoniae*, it enhanced bacterial resistance to macrophages and attenuated organ pathology in mice. This undoubtedly provides a new perspective for future research on sepsis caused by *K. pneumoniae*.

## Data Availability

The datasets presented in this study can be found in online repositories. The names of the repository/repositories and accession number(s) can be found in the article/Supplementary Material.

## Supplementary material

Supplementary Figure S1 Characteristics and functional analysis of differentially expressed genes (DEGs). **(A)** Unsupervised clustering analysis of DE micro RNAs (miRNAs). Red dots indicate significantly upregulated miRNAs, green dots indicate significantly downregulated miRNAs. **(B)** Volcano plots of miRNAs. Red dots indicate upregulated DEmiRNAs, green dots indicate downregulated DEmiRNAs, black dots indicate non-differentially expressed miRNAs.

Supplementary Figure S2 Characteristics and functional analysis of differentially expressed genes (DEGs). **(A)** Unsupervised clustering analysis of the DE messenger RNAs (mRNAs). Red dots indicate significantly upregulated mRNAs, green dots indicate significantly downregulated mRNAs. **(B)** Volcano plots of mRNAs. Red dots indicate upregulated DEmRNAs, green dots indicate downregulated DEmRNAs, black dots indicate non-differentially expressed mRNAs.

Supplementary Figure S3 The HLA-DRB4 expression of mRNA in K.pneumoniae wild-type, ΔygiM and C-ygiM strains. Positive target of HLA-DRB4 was considered to be statistically significant in the infection of K. pneumoniae control, wild type and ΔygiM groups (*, p<0.05, **, p<0.01).
