# Supplementary figures and images for: YgiM may act as a trigger in the sepsis caused by Klebsiella pneumoniae through the membrane-associated ceRNA network
Source: Front Genet. 2022 Sep 23;13:973145. doi: 10.3389/fgene.2022.973145 (PMC9537587; doi:10.3389/fgene.2022.973145)

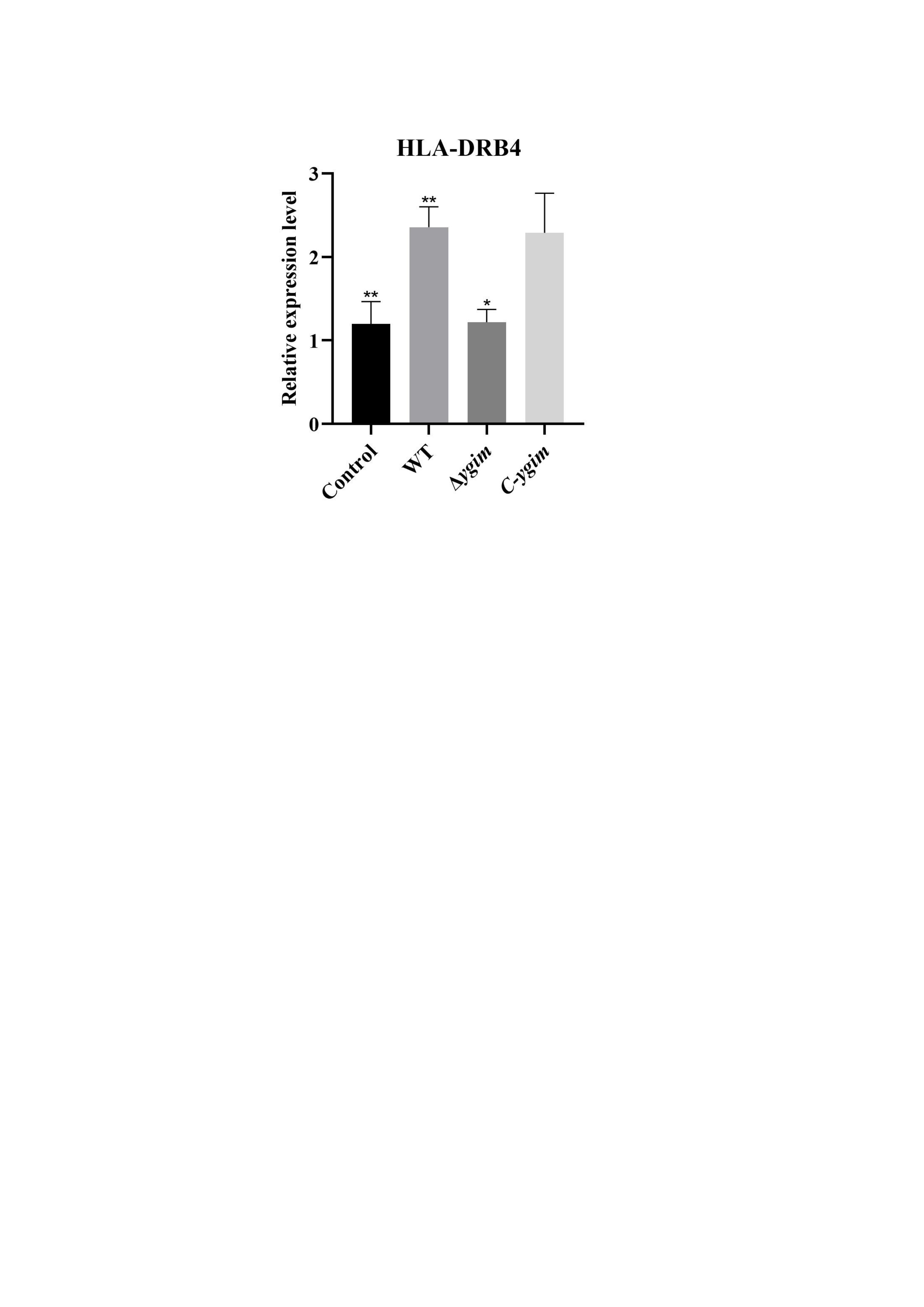

Supplement: Supplementary file 2 [file Image3.JPEG]

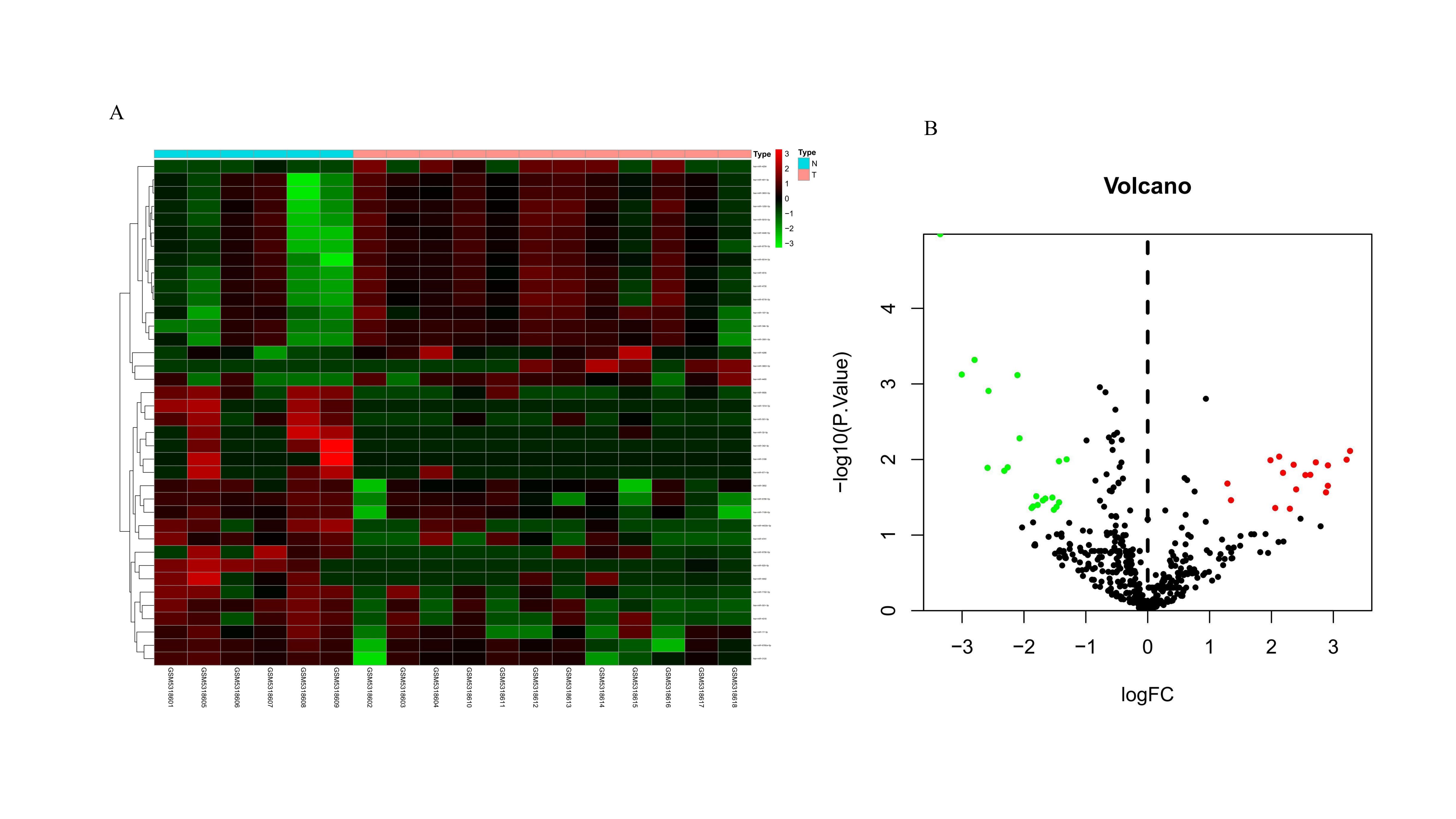

Supplement: Supplementary file 4 [file Image1.JPEG]

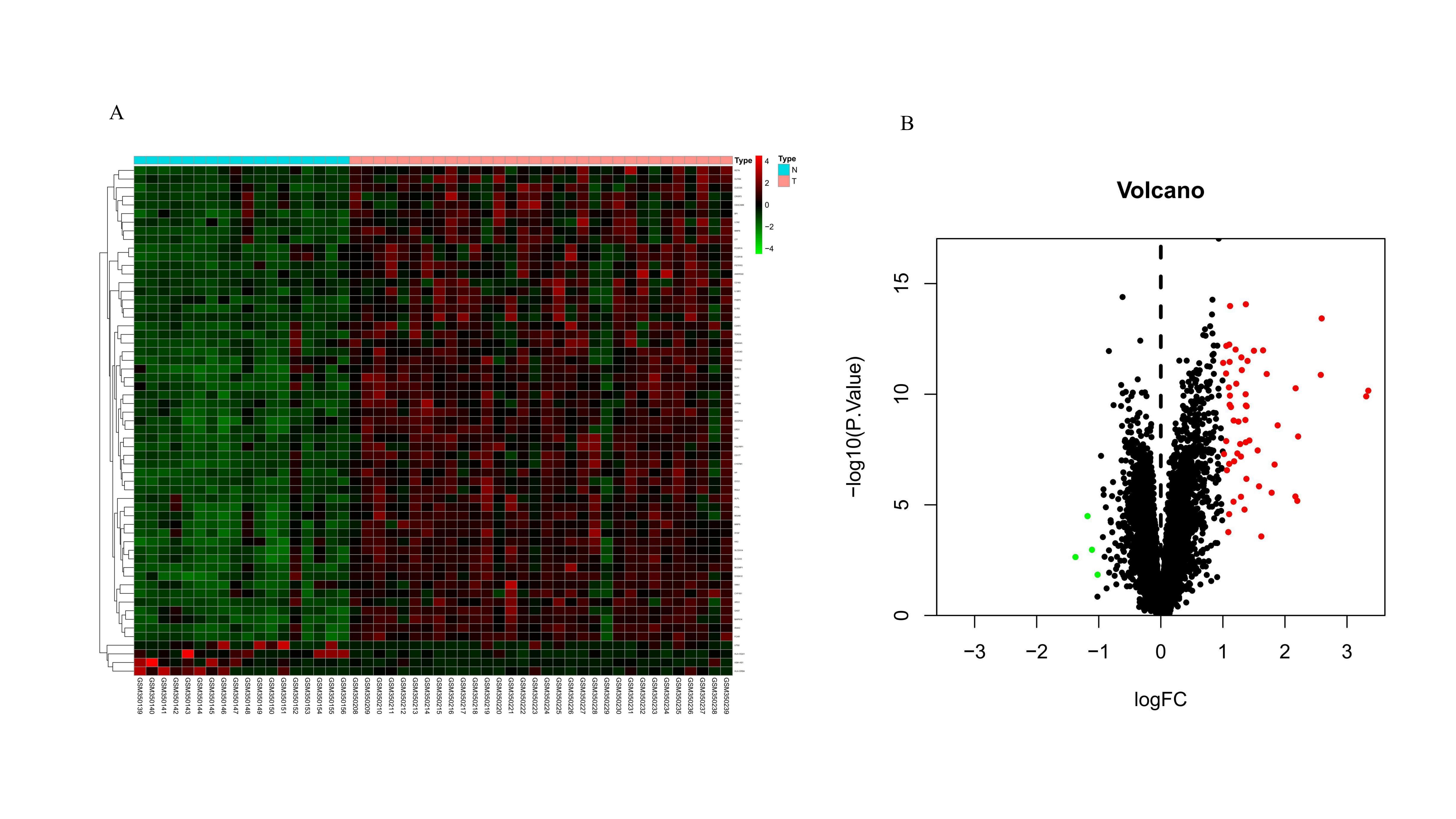

Supplement: Supplementary file 5 [file Image2.JPEG]
